# Carotid Atherosclerosis Progression and Risk of Cardiovascular Events in a Community in Taiwan

**DOI:** 10.1038/srep25733

**Published:** 2016-05-12

**Authors:** Pei-Chun Chen, Jiann-Shing Jeng, Hsiu-Ching Hsu, Ta-Chen Su, Kuo-Liong Chien, Yuan-Teh Lee

**Affiliations:** 1 Clinical Informatics and Medical Statistics Research Center, Chang Gung University, Tao-Yuan 33302, Taiwan; 2 Department of Neurology, Chang Gung University College of Medicine, Chang Gung Memorial Hospital, Chiayi branch, Chiayi 61363, Taiwan; 3 Department of Neurology, National Taiwan University Hospital, Taipei 10002, Taiwan; 4 Department of Internal Medicine, National Taiwan University Hospital, Taipei 10002, Taiwan; 5 Insitute of Occupational Medicine and Industrial Hygiene, National Taiwan University, Taipei 10055, Taiwan; 6 Institute of Epidemiology and Preventive Medicine, National Taiwan University, Taipei 10055, Taiwan

## Abstract

The authors investigated the association between progression of carotid atherosclerosis and incidence of cardiovascular disease in a community cohort in Taiwan. Data has rarely been reported in Asian populations. Study subjects were 1,398 participants who underwent ultrasound measures of common carotid artery intima-media thickness (IMT) and extracranial carotid artery plaque score at both 1994–1995 and 1999–2000 surveys. Cox proportional hazards model was used to assess the risk of incident cardiovascular disease. During a median follow-up of 13 years (1999–2013), 71 strokes and 68 coronary events occurred. The 5-year individual IMT change was not associated with development of cardiovascular events in unadjusted and adjusted models. Among subjects without plaque in 1994–1995, we observed elevated risk associated with presence of new plaque (plaque score >0 in 1999–2000) in a dose-response manner in unadjusted and age- and sex- adjusted models. The associations attenuated and became statistically non-significant after controlling for cardiovascular risk factors (hazard ratio [95% confidence interval] for plaque score >2 vs. 0: stroke, 1.61 [0.79–3.27], coronary events, 1.13 [0.48–2.69]). This study suggested that carotid plaque formation measured by ultrasound is associated increased risk of developing cardiovascular disease, and cardiovascular risk factors explain the associations to a large extent.

Ultrasound measures of carotid intima-media thickness (IMT) and plaque are non-invasive methods for measuring atherosclerotic burden and strongly associated with vascular risk factors^1^ and incidence of cardiovascular events^2^,^3^. Carotid IMT and plaque have been used as a surrogate of cardiovascular diseases in observational studies and clinical trials, in which change of these carotid ultrasound measurements over time were the outcome measures for assessing the effects of exposures or interventions^4^,^5^. However, existing data regarding the association between progression of carotid IMT and risk of cardiovascular events remains inconclusive^6^,^7^,^8^. A recent meta-analysis collating data from cohort studies of general populations revealed no relationship between progression rate of common carotid artery intima-media thickness (CCA IMT) and risk of cardiovascular events^6^. Although the analysis was on a large scale, the populations included were mainly from US and Europe; there was only 1 stroke event from Chinese participants. Furthermore, evidence showed that single measurement of carotid plaque is a stronger predictor of risk of cardiovascular events than is IMT^9^,^10^; the risk associated with plaque progression, however, has not been well documented yet in general populations.

Evidence has shown ethnic differences in cardiovascular risk factors and atherosclerosis process^11^,^12^. Whether the observations derived mainly from studies of Caucasians origin holds true in Asian populations is unclear. Within a community cohort in Taiwan, we investigated whether change of CCA IMT and formation of new carotid plaque is associated with risk of developing cardiovascular diseases.

## Results

### Change of mean CCA IMT and cardiovascular events

Table 1 shows the baseline characteristics in 1994–1995 examination of subjects in each quartile of CCA IMT rate-of-change. Of all subjects, men accounted for 43.9%. The mean age was 55.9 years old (s.d. 10.5 years). The median level of CCA IMT rate-of-change per year was 0.016 mm, with an interquartile range of −0.008 to 0.036 mm; the mean (SD) was 0.014 (0.041) mm. Subjects in the lowest quartile were older and had greater mean CCA IMT in 1994–1995. Individuals in the lowest and highest quartiles exhibited higher levels of blood pressure and lower levels of high-density lipoprotein cholesterol. Subjects with greater increase in CCA IMT were more likely to have family history of heart disease.

During a median follow-up period of 13.4 years, 71 strokes and 68 coronary events occurred, and 134 individuals had combined endpoints. The incidence rates and HRs of the cardiovascular events are shown in Table 2. In all unadjusted and multivariable-adjusted models, change in CCA IMT was not associated with risk of developing any cardiovascular events. In model 4, the adjusted HR (95% CI) per one SD increase in change of CCA IMT was 0.95 (0.83–1.09) for combined endpoint, 0.97 (0.81–1.16) for stroke, and 0.95 (0.78–1.15) for coronary events.

### Formation of carotid artery plaque and cardiovascular events

During the 5-year period between the two ultrasound scans, 255 (20.5%) subjects developed new carotid artery plaques (Table 3). Men accounted for 50.6% of subjects with new plaques and 39.7% of those who sustained without plaques (p < 0.001). Individuals with plaque presence were older, and more likely to be smokers and to have hypertension and diabetes. The formation of new plaque was also associated with greater body mass index, waist and hip circumference and unfavorable lipid levels.

Of 1241 subjects, 57 strokes and 49 coronary events occurred, and 103 individuals had combined endpoints (Table 4). The incidence rate of each of the three endpoints was 2–3 times higher in subjects developing new plaque than in subjects without plaque. The unadjusted HR (95% CI) in association with presence of new plaque was 2.26 (1.51–3.38) for combined endpoint, 2.51 (1.48–4.28) for stroke and 1.95 (1.07–3.55) for coronary events. There was no longer an association after controlling for age, sex and cardiovascular risk factors (models 1 to 3, Table 4). The incidence rate of cardiovascular events increased with increasing plaque score. The unadjusted HR (95% CI) for plaque score <2 comparing with no plaque was 3.29 (2.03–5.33) for combined endpoint, 3.81 (2.05–7.08) for stroke and 2.65 (1.27–5.54) for coronary events. The association attenuated by controlling for age and sex but remained statistically significant (model 1 of Table 4, p for trend across groups of plaque score: 0.031 for combined endpoint, 0.044 for stroke, and 0.23 for coronary events). The HRs became statistically non-significant after additionally adjusting for cardiovascular risk factors (models 2 and 3) for all three endpoints.

## Discussion

In the present cohort study, progression rate of CCA IMT was not associated with subsequent risk of cardiovascular events. We found that the presence of new plaque was associated with increased incidence of cardiovascular events in a dose-response manner. However, cardiovascular risk factors explain the associations to a large extent.

In line with our observations, in an individual meta-analysis including 16 general populations, Lorenz *et al.* reported no association between yearly progression of CCA IMT and subsequent risk of cardiovascular disease^6^. This observation is consistent with accumulating evidence suggesting that ultrasound measure of CCA IMT, although more accurate than IMT measurements of other carotid sites, does not directly reflect atherosclerotic process. Increased IMT mainly represents hypertensive hypertrophy of the media layer and is substantially influenced by genetic factors^9^,^13^,^14^. In addition, plaque rarely occurs in CCA but is more prevalent in internal carotid artery and bifurcation^9^. A recent study has shown that IMT progression determined by multiple carotid segments but not by CCA alone predicted vascular events^15^. A meta-analysis also revealed improved accuracy for predicting risk of coronary events when IMT measurement consisted of not only CCA but also other segments, in which plaques are more prevalent, suggesting the importance of incorporating plaque assessment in extracranial carotid artery for risk prediction^10^.

A substantial amount of epidemiological evidence has shown that quantification of plaque burden, measured by ultrasonography, is a stronger predictor of cardiovascular endpoints than is IMT^9^,^10^,^16^,^17^. However, limited data is available on the association between plaque progression and cardiovascular outcomes. To the best of our knowledge, two epidemiological studies to date have been carried out in this area. Both studies were performed in patients with vascular disease^13^,^18^. In these studies, progression of plaque, measured using plaque area^13^ and total plaque volume^18^, was associated increased risk of cardiovascular events. This is in contrast to our observations, which showed that after controlling for cardiovascular risk factors, formation and extent of new plaque was not associated with risk of coronary events, and the association with stroke weakened substantially. Discrepancies in the results could reflect the different methodologies used. Our study included general populations of Chinese ethnicity, whereas participants of previous studies were patients attending vascular prevention clinics in Ontario, Canada^13^,^18^, where the most common ethnic origin is European origin. Furthermore, differences in quantitative measures of plaques and artery segments may explain the inconsistencies in results. Wannarong *et al.* reported that progression of plaque volume was more strongly associated with increased incidence of cardiovascular events than was progression of plaque area^18^. In the Multi-Ethnic Study of Atherosclerosis, which included 6 metrics of carotid plaque, only presence of plaque defined by ≥25% diameter narrowing was associated with increased risk of stroke^19^. However, in that study progression of plaque was not assessed. Further studies focus on comparison of various measures of plaque progression may help clarify this issue.

Our analysis in the community cohort study provided data from Asia. Although the multiethnic study has included Chinese^7^, ethnic-specific HR was not reported perhaps because of small sample sizes. We evaluated individual progression of carotid atherosclerosis during a period of 5 years, which is a fairly long duration that has been suggested adequate for accumulating sufficient number of incident cases of ultrasound measured carotid stenosis^20^.

There are a number of limitations to this study. First, measurement errors may occur as we used only two ultrasound scans to determine change of CCA IMT. Our analysis showed that subjects in the lowest quartile of CCA IMT change had the greatest CCA IMT in 1994–1995 visit (Table 1), suggesting that “regression to the mean”, a phenomenon resulting from random measurement error^21^, might occur and partly explain the CCA IMT change. The measurement error might obscure relationships between CCA IMT change and cardiovascular events, and might be a possible explanation of no association between known cardiovascular risk factors and CCA IMT progression (Table 1). Furthermore, in the general population at low risk, change of CCA IMT was relatively small. The measurement variability may be substantial, resulting in decreased power of the study. Second, we assessed presence and extent of new plaques using solely plaque score. Other metrics of plaque progression may be more powerful in assessment of risk of cardiovascular events. Third, half of survived members of the cohort were excluded from our analysis, which may influence the generalizability of results to the original cohort. In addition, the value of carotid artery measurements and the estimated incidence of cardiovascular events of the study subjects might be lower than that of the original cohort, as excluded subjects were older and more likely to be smokers. However, the effect estimates, i.e., HRs, were less likely to be biased if underestimation of the event incidence is assumed to be of similar magnitude across groups of carotid artery measurements. Fourth, the statistical power might be insufficient to detect a moderate association in categories of plaque score with limited number of study subjects. Last, multiple hypothesis tests were conducted to assess the associations between carotid artery measurements and multiple outcomes. Chance of false positive findings can increase as the number of multiple tests increases. Results of our analyses, particularly those for stroke and coronary events separately, were thus considered exploratory rather than confirmatory.

In this community cohort study in Taiwan, carotid atherosclerosis progression, measured by plaque formation but not IMT change, is associated with risk of developing cardiovascular disease. Adjusting for cardiovascular risk factors substantially weakened the associations, suggesting that the presence and extent of carotid plaque could reflect underlying cardiovascular risks. Formation of carotid plaque may be used as a “red flag” signaling high risk of an individual and a surrogate of risk of cardiovascular events for assessment of health interventions.

## Methods

### Study participants

The Chin-Shan Community Cardiovascular Cohort Study (CCCC) is a prospective study of cardiovascular disease began in 1990 in a suburban township 30 km north of Taipei, Taiwan, where people is predominantly ethnic Chinese. CCCC comprises 3602 noninstitutionalized residents aged 35 years old and above who received a health checkup in the baseline survey in 1990 and 1991. Details of the study design and the validity of the data collected has been described elsewhere^22^.

Carotid ultrasonography was performed at the follow-up visits in 1994–1995 and 1999–2000. As of the follow-up visit in 1999–2000, 2971 of the 3602 members of the original cohort were alive, and 1471 subjects received carotid ultrasound examinations at both visits. After excluding individuals with history of stroke and coronary events, 1398 subjects were included in the present study. Individuals excluded from the present analysis were average 2-year older and more likely to be smokers, but did not differ significantly from those included in this study in terms of sex. Study subjects gave informed consent. The study protocol was approved by the institutional review board of the National Taiwan University Hospital, and all methods were performed in accordance with the approved guidelines.

### Carotid artery measurements

We used two indexes to evaluate carotid atherosclerosis: the maximal CCA IMT and the extracranial carotid artery plaque score. These measurements were determined using a Hewlett-Packard SONO 1500 ultrasound system (Palo Alto, CA), equipped with a 7.5-MHz real-time B-mode scanner and a 5.6-MHz pulsed-Doppler mode scanner. All scans were recorded on super-Video home system videotape for subsequent off-line analysis. All of the IMT measurements were done with manual caliper measurement by an experienced technician blinded to subjects’ health status and risk factors. Details of the ultrasonographic methodology, plaque scoring quantified method, and quality control have been reported previously^23^,^24^,^25^. In brief, the maximal CCA IMT was measured bilaterally on the far wall of the CCA at sites 0 to 10 mm and 10 to 20 mm, respectively, distal from the bulb bifurcation (i.e., the beginning of the bulbar widening). Plaques were avoided in the measurement of IMT. The maximal IMT values at the 4 CCA sites, 2 each from the right and the left arteries, were then used to calculate the mean of maximal IMT. The plaque measurements were examined bilaterally for the following carotid artery segments: proximal CCA and distal CCA (≥20 and <20 mm proximal to the bulb bifurcation, respectively), bulb, internal carotid artery, and external carotid artery. A focal thickening of IMT with >50% of thickness than adjacent IMT was considered as an atherosclerotic plaque. The presence and extent of atherosclerosis was graded for each chosen segments: grade 0, normal or no observable plaque; grade 1, one small plaque (diameter stenosis of <30%); grade 2, one medium plaque (30% to 49% diameter stenosis) or multiple small plaques, grade 3, one large plaque (50% to 99% diameter stenosis) or multiple plaques with at least one medium plaque, and grade 4, total occlusion. The grades from all segments were then summed up to obtain the plaque score^24^. The within- and between-observer correlation coefficients were 0.86 to 0.93 and 0.70 to 0.87, respectively, for the CCA IMT measurements. Evaluation of the reproducibility of the plaque grade showed good reproducibility with a Kappa statistic of 0.70^23^,^24^.

We calculated the change of mean CCA IMT by subtracting the mean IMT measured in the follow-up survey in 1994–1995 from that measured in 1999–2000 visit. The rate of change per year was then calculated by using change of mean CCA IMT divided by the time interval between the two ultrasound scans (approximately 5 years). A plaque score of zero indicates absence of plaque.

### Cardiovascular risk factors

Methods of in-person questionnaire interviews, blood processing, and laboratory measurements have been reported elsewhere^22^,^26^. We used data collected in 1994–1995 and 1999–2000 visits. Hypertension was defined as the systolic blood pressure of 140 mmHg or higher, or the diastolic blood pressure of 90 mmHg or higher, or receiving antihypertensive medication^25^. Individuals with a fasting serum glucose level at or above 126 mg/dl or history of hypoglycaemic medication were considered as having diabetes mellitus.

### Outcomes

The study outcomes were stroke, coronary events and the combined endpoint (stroke and coronary events). Procedures for our documentation of the cardiovascular events have been described and validated^22^,^27^. Events were ascertained by reviewing medical records and official death certificates, and by household visits at approximately 2-year cycles to inquire about all interim hospitalizations for the cardiovascular events. Stroke was defined as a sudden neurologic deficit of vascular origin persisting longer than 24 hours with supporting imaging data. Transient ischemic attacks were not included. Coronary events include coronary death, nonfatal myocardial infarction, and a hospital admission for procedures of coronary artery bypass graft and percutaneous coronary intervention. These endpoints were reviewed and confirmed by cardiologists. In the present analysis, we included the outcome events occurring during the follow-up period starting from dates of 1999–2000 visits until the end of 2013.

### Statistical analysis

We used two CCA IMT variables representing change of CCA IMT: the quartiles of the rate of change per year and per one SD difference of CCA IMT change. In the analyses of carotid plaque, we included 1241 subjects without any plaque, i.e., plaque score = 0, in 1994–1995. The plaque score in 1999–2000 was defined as a dichotomous variable representing the presence of new plaque (plaque score ≥1), and further classified into three levels, 0, 1–2, <2. Sociodemographic characteristics and clinical variables between the groups were compared using student t-test or 1-way ANOVA for continuous variables and χ^2^ test for categorical variables.

We used Cox proportional hazards models, which estimated the hazard ratios (HRs) and 95% confidence intervals (CIs), to assess association between progression of carotid atherosclerosis and risk of developing cardiovascular events. For each of the cardiovascular outcomes, we fitted several Cox models with age, sex and cardiovascular risk factors added sequentially to observe whether the association changes by adding the potential confounders. All the variables included in the models are listed in the footnotes of Tables 2 and 4. The confounding variables selected for these models were known risk factors, and based on previous experience with the study cohort and variables with a *P* value less than 0.05 in the univariate analysis. We examined the proportional hazard assumption by including an interaction term of each carotid variable and follow-up time in the unadjusted and adjusted Cox models. The likelihood ratio tests showed that the interactions were not statistically significant, suggesting that the assumption was not violated.

All data analyses were performed using SAS, version 9.3 (SAS Institute Inc., Cary, North Carolina). Level of statistical significance was set at a two-sided *P* value of less than 0.05.

## Additional Information

**How to cite this article**: Chen, P.-C. *et al.* Carotid Atherosclerosis Progression and Risk of Cardiovascular Events in a Community in Taiwan. *Sci. Rep.* **6**, 25733; doi: 10.1038/srep25733 (2016).

## Figures and Tables

**Table 1:** Baseline characteristics in 1994–1995 of participants by quartile of CCA IMT change rate between 1994–1995 and 1999–2000*.

	Total	Range of quartiles (rate-of-change per year, mm)				
		Q1, <−0.008	Q2, −0.008 to <0.016	Q3, 0.016 to <0.036	Q4, >=0.036	P value
	n = 1398	n = 348	n = 355	n = 347	n = 348	
Men, %	43.9	46.3	45.4	39.5	44.3	0.28
Age, years	55.9±10.5	58.5 ± 10.7	55.2 ± 10.6	54.0 ± 10.1	55.8 ± 10.1	<0.001
Current smoker, yes, %	23.3	24.6	23.8	23.6	21.3	0.76
Alcohol drinking, yes, %	22.7	20.4	23.7	23.7	23.1	0.69
Education level, <9 year, %	93.1	94.0	90.4	94.2	93.7	0.16
Body mass index, kg/m^2^	24.2 ± 3.3	24.2 ± 3.6	24.1 ± 3.4	24.6 ± 3.4	24.2 ± 3.0	0.30
Waist circumference, cm	82.3 ± 9.8	83.0 ± 10.1	81.9 ± 9.5	82.1 ± 10.2	82.3 ± 9.4	0.50
Family history of coronary events, yes, %	10.3	8.6	7.9	13.5	11.2	0.056
Hypertension, yes, %	27.9	31.4	24.7	23.6	31.8	0.027
Diabetes, yes, %	15.6	15.8	15.3	16.4	14.9	0.95
Fasting glucose, mg/dL	110.2 ± 28.3	109.8 ± 22.8	108.8 ± 28.2	110.6 ± 32.3	111.7 ± 29.3	0.61
Systolic blood pressure, mmHg	123.6 ± 17.4	124.9 ± 18.0	122.6 ± 17.1	121.2 ± 16.7	125.5 ± 17.5	0.004
Diastolic blood pressure, mmHg	75.9 ± 11.5	76.3 ± 11.4	75.2 ± 11.3	75.6 ± 11.9	76.6 ± 11.3	0.42
High density lipoprotein cholesterol, mg/dL	41.0 ± 11.0	40.2 ± 11.0	42.3 ± 11.4	41.6 ± 10.4	40.0 ± 11.0	0.016
Low density lipoprotein cholesterol, mg/dL	128.7 ± 36.8	130.2 ± 36.4	124.2 ± 36.6	128.6 ± 39.0	131.6 ± 34.7	0.054
Total cholesterol, mg/dL	206.0 ± 43.9	204.9 ± 44.4	202.0 ± 43.5	207.1 ± 44.8	209.9 ± 42.6	0.11
Triglycerides, mg/dL	106.9 ± 93.5	106.4 ± 80.4	99.9 ± 79.5	107.5 ± 95.7	113.7 ± 114.0	0.29
CCA IMT in 1994–1995, mm	0.69 ± 0.19	0.85 ± 0.21	0.69 ± 0.14	0.62 ± 0.13	0.62 ± 0.17	<0.001
CCA IMT in 1999–2000, mm	0.77 ± 0.20	0.67 ± 0.17	0.71 ± 0.14	0.75 ± 0.13	0.93 ± 0.25	<0.001

Abbreviations: CCA IMT, common carotid artery intima-media thickness.

^*^Values are mean ± s.d. (standard deviation) unless otherwise indicated.

**Table 2:** Hazard ratios of endpoints during follow-up period in relation to CCA IMT progression between 1994–1995 survey and 1999–2000 survey.

	Range of quartiles (rate-of-change per year, mm)					
	Q1 <−0.008	Q2 −0.008 to <0.016	Q3 0.016 to <0.036	Q4 >=0.036	*p* for trend	Per 1 SD increase
Combined endpoint						
No. of events/person-years	40/4002	33 /4254	21/4291	40/4021		
Incidence rate (per 1000 person-years)	10.0	7.8	4.9	9.9		
Hazard ratio (95% confidence interval)						
Unadjusted model	1.0	0.78 (0.49–1.24)	0.50 (0.29–0.84)	0.99 (0.64–1.53)	0.60	0.92 (0.77–1.09)
Model 1	1.0	0.91(0.57–1.44)	0.64(0.38–1.09)	1.14(0.73–1.76)	0.85	0.95(0.82–1.10)
Model 2	1.0	0.96 (0.61–1.53)	0.69(0.41–1.18)	0.99(0.63–1.55)	0.70	0.91(0.80–1.04)
Model 3	1.0	1.16 (0.71–1.91)	0.68 (0.38–1.22)	1.09 (0.68–1.75)	0.93	0.95 (0.82–1.09)
Model 4	1.0	1.08 (0.68–1.72)	0.77 (0.45–1.32)	1.09 (0.70–1.71)	0.95	0.95 (0.83–1.09)
Stroke						
No. of events/person-years	20/4092	19/4320	10/4376	22/4147		
Incidence rate (per 1000 person-years)	4.9	4.4	2.3	5.3		
Hazard ratio (95% confidence interval)						
Unadjusted model	1.0	0.90 (0.48–1.69)	0.47 (0.22–1.01)	1.08 (0.59–1.98)	0.85	0.92 (0.73–1.17)
Model 1	1.0	1.07(0.57–2.01)	0.61(0.28–1.30)	1.25(0.68–2.28)	0.75	0.95(0.78–1.16)
Model 2	1.0	1.12(0.60–2.11)	0.66(0.31–1.41)	1.11(0.60–2.05)	0.96	0.92(0.77–1.09)
Model 3	1.0	1.11 (0.57–2.18)	0.67 (0.30–1.49)	1.22 (0.65–2.31)	0.79	0.95 (0.79–1.15)
Model 4	1.0	1.30 (0.69–2.48)	0.73 (0.34–1.58)	1.25 (0.67–2.32)	0.76	0.97 (0.81–1.16)
Coronary events						
No. of events/person-years	21/4120	15/4348	12/4340	20/4130		
Incidence rate (per 1000 person-years)	5.1	3.4	2.8	4.8		
Hazard ratio (95% confidence interval)						
Unadjusted model	1.0	0.68 (0.35–1.32)	0.55 (0.27–1.12)	0.95 (0.51–1.75)	0.70	0.93 (0.73–1.19)
Model 1	1.0	0.77(0.39–1.49)	0.71(0.35–1.44)	1.08(0.58–1.99)	0.91	0.96(0.78–1.19)
Model 2	1.0	0.81(0.42–1.58)	0.76(0.37–1.56)	0.93(0.50–1.73)	0.75	0.92(0.77–1.11)
Model 3	1.0	0.94 (0.46–1.95)	0.68 (0.30–1.52)	0.97 (0.50–1.88)	0.76	0.93 (0.76–1.13)
Model 4	1.0	0.92 (0.47–1.80)	0.83 (0.40–1.72)	1.00 (0.54–1.88)	0.94	0.95 (0.78–1.15)

Abbreviations: CCA IMT, common carotid artery intima-media thickness.

Models included the following variables: model 1, age (per unit) and sex; model 2, all model 1 variables plus mean value of common carotid intima-media thickness of 1994–1995 visit and 1999–2000 visit; model 3, all model 2 variables plus cardiovascular risk factors measured in 1994–1995 visit, including current smoking (yes or no), waist circumference (per unit), diabetes (yes or no), hypertension (yes or no), and low density lipoprotein cholesterol (per unit) (smoking and lipid parameters were not included when modeling risk of stroke); and model 4, all model 2 variables, smoking, plus mean value of cardiovascular risk factors (per unit) in 1994–1995 visit and 1999–2000 visit, including waist circumference, fasting glucose, systolic blood pressure, and low density lipoprotein cholesterol (smoking was not included when modeling risk of stroke).

**Table 3:** Baseline characteristics of participants without plaque in 1994–1995 by presence of new plaque in 1999–2000*.

	New plaque in 1999–2000		*P* value
	No	Yes	
	n = 986	n = 255	
Men, %	39.7	50.6	0.002
Age, years	53.2 ± 9.7	60.2 ± 9.5	<0.001
Current smoker, yes, %	20.8	27.6	0.021
Alcohol drinking, yes, %	22.9	23.1	0.95
Education level, <9 year, %	92.4	94.1	0.34
Body mass index, kg/m^2^	24.2 ± 3.3	24.7 ± 3.3	0.048
Waist circumference, cm	81.5 ± 9.8	84.4 ± 9.3	<0.001
Family history of coronary events, yes, %	11.2	9.4	0.42
Hypertension, yes, %	23.7	35.2	<0.001
Diabetes, yes, %	13.0	24.1	<0.001
Fasting glucose, mg/dL	107.8 ± 22.7	118.2 ± 42.6	<0.001
Systolic blood pressure, mmHg	121.1 ± 16.1	129.0 ± 18.1	<0.001
Diastolic blood pressure, mmHg	75.2 ± 11.3	78.4 ± 11.1	<0.001
High density lipoprotein cholesterol, mg/dL	41.5 ± 11.0	40.0 ± 10.8	0.056
Low density lipoprotein cholesterol, mg/dL	125.9 ± 35.7	137.5 ± 38.9	<0.001
Total cholesterol, mg/dL	203.4 ± 42.4	216.4 ± 47.3	<0.001
Triglycerides, mg/dL	103.6 ± 85.7	121.2 ± 124.6	0.036
CCA IMT in 1994–1995	0.64 ± 0.14	0.74 ± 0.17	<0.001
CCA IMT in 1999–2000	0.71 ± 0.15	0.90 ± 0.21	<0.001

Abbreviations: CCA IMT, common carotid artery intima-media thickness.

^*^Values are mean ± s.d.(standard deviation) unless otherwise indicated.

**Table 4:** Hazard ratios of endpoints during follow-up period in relation to presence of new carotid plaque among subjects without plaque in 1994–1995.

	Formation of plaque in 1999–2000		Plaque score in 1999–2000			
	no	yes	0	1–2	>2	P for trend
Combined endpoint						
No. of events/person-years	66/12074	37/2917	66/12074	15/1736	22/1180	
Incidence rate (per 1000 person-years)	5.5	12.7	5.5	8.6	18.6	
Hazard ratio (95% confidence interval)						
Unadjusted model	1.0	2.26 (1.51–3.38)	1.0	1.55 (0.88–2.71)	3.29 (2.03–5.33)	<0.001
Model 1	1.0	1.43 (0.93–2.19)	1.0	1.11 (0.63–1.96)	1.84 (1.09–3.09)	0.031
Model 2	1.0	1.01 (0.63–1.63)	1.0	0.74 (0.38–1.41)	1.37 (0.78–2.41)	0.48
Model 3	1.0	1.09 (0.71–1.67)	1.0	0.80 (0.44–1.43)	1.45 (0.86–2.40)	0.29
Stroke						
No. of events/person-years	35/12289	22/3001	35/12289	8/1760	14/1241	
Incidence rate (per 1000 person-years)	2.8	7.3	2.8	4.5	11.3	
Hazard ratio (95% confidence interval)						
Unadjusted model	1.0	2.51 (1.48–4.28)	1.0	1.57 (0.73–3.39)	3.81 (2.05–7.08)	<0.001
Model 1	1.0	1.53 (0.87–2.70)	1.0	1.07 (0.49–2.34)	2.10 (1.08–4.10)	0.044
Model 2	1.0	1.30 (0.72–2.36)	1.0	1.04 (0.47–2.30)	1.61 (0.79–3.27)	0.23
Model 3	1.0	1.24 (0.70–2.20)	1.0	0.85 (0.38–1.89)	1.71 (0.88–3.32)	0.18
Coronary events						
No. of events/person-years	33/12315	16/2999	33/12315	7/1758	9/1241	
Incidence rate (per 1000 person-years)	2.7	5.3	2.7	4.0	7.3	
Hazard ratio (95% confidence interval)						
Unadjusted model	1.0	1.95 (1.07–3.55)	1.0	1.46 (0.65–3.30)	2.65 (1.27–5.54)	0.010
Model 1	1.0	1.36 (0.73–2.55)	1.0	1.14 (0.50–2.60)	1.64 (0.75–3.59)	0.23
Model 2	1.0	0.82 (0.40–1.68)	1.0	0.59 (0.22–1.59)	1.13 (0.48–2.69)	0.95
Model 3	1.0	0.92 (0.48–1.74)	1.0	0.68 (0.28–1.63)	1.22 (0.56–2.64)	0.84

Models included the following variables: model 1, age (per unit) and sex; model 2, all model 1 variables plus cardiovascular risk factors measured in 1994–1995 visit, including current smoking (yes or no), waist circumference (per unit), diabetes (yes or no), hypertension (yes or no), and low density lipoprotein cholesterol (per unit) (smoking and lipid parameters were not included when modeling risk of stroke); and model 3, all model 1 variables, smoking, plus mean value of cardiovascular risk factors (per unit) in 1994–1995 visit and 1999–2000 visit, including waist circumference, fasting glucose, systolic blood pressure, and low density lipoprotein cholesterol (smoking was not included when modeling risk of stroke).
